# ATP P2X3 receptors and neuronal sensitization

**DOI:** 10.3389/fncel.2013.00236

**Published:** 2013-12-04

**Authors:** Elsa Fabbretti

**Affiliations:** University of Nova Gorica, Center for Biomedical Sciences and EngineeringNova Gorica, Slovenia

**Keywords:** trigeminal neurons, pain, receptor plasticity, purinergic signaling, migraine

## Abstract

Increasing evidence indicates the importance of extracellular adenosine triphosphate (ATP) in the modulation of neuronal function. In particular, fine control of ATP release and the selective and discrete ATP receptor operation are crucial elements of the crosstalk between neuronal and non-neuronal cells in the peripheral and central nervous systems. In peripheral neurons, ATP signaling gives an important contribution to neuronal sensitization, especially that involved in neuropathic pain. Among other subtypes, P2X3 receptors expressed on sensory neurons are sensitive even to nanomolar concentrations of extracellular ATP, and therefore are important transducers of pain stimuli. P2X3 receptor function is highly sensitive to soluble factors like neuropeptides and neurotrophins, and is controlled by transduction mechanisms, protein-protein interactions and discrete membrane compartmentalization. More recent findings have demonstrated that P2X3 receptors interact with the synaptic scaffold protein calcium/calmodulin-dependent serine protein kinase (CASK) in a state dependent fashion, indicating that CASK plays a crucial role in the modulation of P2X3 receptor stability and efficiency. Activation of P2X3 receptors within CASK/P2X3 complex has important consequences for neuronal plasticity and possibly for the release of neuromodulators and neurotransmitters. Better understanding of the interactome machinery of P2X3 receptors and their integration with other receptors and channels on neuronal surface membranes, is proposed to be essential to unveil the process of neuronal sensitization and related, abnormal pain signaling.

## Introduction

### Adenosine triphosphate as a chemical inducer of sensitization

Sensitization is a process whereby primary sensory neuron afferents and central synapses become hyper-responsive to extracellular nociceptive stimuli so that they underlie neuropathic and chronic pain, including allodynia, hyperalgesia and spontaneous pain. Peripheral and central sensitization are thought to be supported by enhanced release of neurotransmitters and peptides, often co-released with adenosine triphosphate (ATP), from primary afferents to spinal synapses (Bardoni et al., 1997). Activation of the corresponding receptors in postsynaptic dorsal horn neurons induces central sensitization.

Fast conductive myelinated A*δ* fibers and slow non-myelinated C-fibers sense different stimuli, in particular mechanical/chemical or tactile stimuli (Basbaum et al., 2009). Whether A- or C-fibers are more important for the generation of spontaneous firing in neuropathic pain, remains an unanswered question. One important priority for translational medicine is the identification of biomarkers for the functional role of distinct classes of C-fibers and A*δ* fibers and for their transition from mono- to poly-modal function in chronic pain. It is not excluded that cellular crosstalk at ganglion level might also induce functional plasticity in non-nociceptive neurons to be recruited in persistent allodynia (Ueda, 2008). The recruitment of non-nociceptive sensory fibers generates an additional level of complexity that renders the sensitization incompletely understood in its complex molecular constituents and temporal evolution, with consequent slow development of new drugs to prevent/revert it.

One important consideration regards the differential contribution of sensory fibers in humans and rodents and, therefore, the difficulties to apply experimental data to clinically-useful models. Experiments performed with infrared diode laser stimulation on human subjects affected by painful neuropathies have demonstrated that pain conditions are associated with impaired function of A*δ* fibers and low involvement of un-myelinated C-fibers (Tzabazis et al., 2011; Moeller-Bertram et al., 2013), while the opposite is found in rodents (Shields et al., 2010; Zhang et al., 2013). Nonetheless, a species-dependent difference in neural substrates of pain, as recently found in P2X3 receptor sequence (Serrano et al., 2012; Sundukova et al., 2012), does not exclude similar chemo-transduction mechanisms based on analogous mediators and modulators.

The molecular basis of transitions from acute sensitization to long-term hypersensitivity relies on complex temporal and spatial molecular mechanisms that are primed by exposure to soluble factors and intracellular neuronal and non-neuronal signaling. Gene expression and protein trafficking then strongly contribute to change pain receptor expression, supporting dysfunctional action potential firing into aberrant neurotransmitter release at the presynaptic terminal and, thus, inducing central sensitization of spinal and brainstem networks.

Among the soluble and cellular factors responsible for the early molecular signature of fiber sensitization and spontaneous aberrant firing in a variety of pain-related diseases, one powerful candidate molecule is extracellular ATP (Hamilton and McMahon, 2000), co-released with other neurotransmitters and peptides or after mechanical stress by a number of different mechanisms (Corriden and Insel, 2010; Novak, 2011). Indeed, ATP acute injection activates C-nociceptors in healthy human skin without the involvement of mechano-responsive or mechano-insensitive C-fibers (Hilliges et al., 2002). ATP (whose extracellular concentration is limited in time and space by ectonucleotidases that generate active metabolites) binds to different subtypes of ligand-gated P2X channels or metabotropic P2Y receptors (Burnstock, 2008), amplifying the spectrum of reactive molecules in the extracellular space (Browne and North, 2013).

Combinatorial expression of ATP receptors with different affinity for ATP in distinct cell types allows modulation of purinergic signaling in different tissues. Primary sensory neurons widely express P2X3 receptors (Vulchanova et al., 1998) sensitive to nanomolar ATP concentrations (Sokolova et al., 2006) and implicated in the modulation of pain sensitivity as demonstrated using P2X3 knockout (KO) mice (Cockayne et al., 2000; Souslova et al., 2000; Zhong et al., 2001; Cockayne et al., 2005). Recent pharmacological research has been directed to discover new drugs capable of inhibiting P2X3 receptors because their pharmacological block could provide a significant contribution to reduce inflammatory and neuropathic pain (Ford, 2012; North and Jarvis, 2013). Nevertheless, only a few P2X3-selective antagonists have been reported to date (Jarvis et al., 2002; Ford, 2012) and are currently undergoing clinical trials (Fabbretti and Nistri, 2012).

While it is well known that changes in the activity of voltage-gated ion channels expressed by sensory neurons can contribute to chronic pain sensitization (McCleskey and Gold, 1999), the focus of the present review is on ATP-mediated signaling since it represents an early chemical signal that triggers pain in normal circumstances and that can predate the establishment of neuronal sensitization (Hamilton and McMahon, 2000). ATP, working through different (yet unknown) plasticity processes, eventually confers novel maladaptive activity to neurons and non-neuronal cells in the entire tissue. Together with ATP, several soluble factors and neuropeptides like nerve growth factor (NGF), calcitonin gene-related peptide (CGRP), cytokines and prostaglandins cooperate either to directly activate nociceptors (as well as to induce secondary long-lasting chain of genomic changes) or to evoke indirect paracrine responses after non-neuronal cells activation (Shu and Mendell, 2001; Giniatullin et al., 2008; Jakobsson, 2010; Kuner, 2010; Cady et al., 2011).

## Role of ATP-gated P2X3 receptors in neurogenic inflammation and neuronal sensitization

Inflammatory mediators influence neuronal expression of nociceptors and ion channels including ATP receptors, therefore contributing to spontaneous activity of sensory fibers and closing a vicious circle of pathological hyper-responsiveness (Ellis and Bennett, 2013).

Neuronal/non-neuronal cell crosstalk is highly modulated by neuronal ATP and its action not only on P2X3 receptors but also on low affinity ATP receptors (P2X4 or P2X7) known to give a strong contribution in inflammatory response (Toulme et al., 2010; Inoue and Tsuda, 2012). In addition, the reactivity of resident microglia-like cells (macrophages) in ganglia (Villa et al., 2010; Franceschini et al., 2013a) opens new vistas on the cellular mechanisms of regulation of neuronal sensitization at ganglion level.

The inflammatory components of neuropathic pain include activation of toll-like receptors (TLR) on neurons and non-neuronal cells (Christianson et al., 2011; Stokes et al., 2013). Experimental TLR stimulation with the component of the bacterial wall lipopolysaccharide (LPS) promotes significant up-regulation of P2X3 receptor function with faster recovery from desensitization (Franceschini et al., 2013b). This treatment also facilitates release of ATP (Franceschini et al., 2012) and tumor necrosis factor alpha (TNF*α*; Franceschini et al., 2013a). These data suggest that, in sensory ganglion culture, the development of a neuroinflammatory profile facilitates the release of endogenous mediators (including ATP and cytokines) to reinforce the activation of inflammatory cells and constitutively potentiates P2X3 receptors to amplify nociceptive signaling. Similar purinergic signaling likely occurs at central synapse, where block of ATP could represent a potential therapeutic target to limit microglia-mediated inflammatory responses associated with chronic pain sensitization (Ulmann et al., 2008; Jakobsson, 2010). The possibility of ATP-mediated crosstalk also within ganglia has recently been proposed (Ceruti et al., 2008; Ohara et al., 2009; Belzer et al., 2010; Ceruti et al., 2011; Huang et al., 2013), supporting the intrinsic role of satellite glial cells for adaptation mechanisms during chronic pain (Hanani, 2012; Kung et al., 2013) and their role as inflammatory cells (van Velzen et al., 2009).

These observations suggest that there is a complex sequence of cellular responses that exert chemical tissue priming to create the basal conditions permissive for sensitization. In analogy with adaptive immune responses, we expect that interleukin (IL-1*β*) priming causes amplification of antigen-presenting cells in ganglia, in particular satellite glial cells (Ben-Sasson et al., 2011).

## Intracellular signaling induces sensitization via P2X3 receptor upregulation

A major property of P2X3 receptors is the ability to rapidly adapt their function to changes in the extracellular milieu via receptor redistribution, trafficking, and phosphorylation. Our former studies have demonstrated that P2X3 receptors of trigeminal sensory neurons are tightly controlled by the fine balance between kinases and phosphatases, which regulate even the basal operational activity of these receptors (Giniatullin et al., 2008).

NGF is sufficient to directly sensitize nociceptive endings causing spontaneous pain (Bennett et al., 1998; Shu and Mendell, 2001; Rukwied et al., 2013), to sensitize P2X3 expressing nociceptors in mice (Ramer et al., 2001; D’Arco et al., 2007, 2009) and to induce acute sensitization of nociceptors in man (McKelvey et al., 2013; Silberstein, 2013). Manipulating NGF levels produces a major impact on ATP-mediated responses by altering intraneuronal signaling pathways (D’Arco et al., 2007; Giniatullin et al., 2008). Pharmacological blockade of protein kinase C (PKC) or Calcium/calmodulin-dependent protein kinase II (CamKII) activation prevents NGF-induced sensitization (Bonnington and McNaughton, 2003), and NGF neutralization unleashes the Sarcoma tyrosine kinase (Src) kinase blocker C-terminal Src kinase (Csk) to limit P2X3 receptor function at membrane level (D’Arco et al., 2009) and to inhibit neuronal sensitization (Liu et al., 2008). cAMP response element binding protein (CREB)-mediated gene expression in dorsal horn neurons establishes peripheral and central sensitization (Fang et al., 2002) suppressed by extracellular signal-regulated kinase (ERK) blockers, and by protein kinase A (PKA), PKC or CaMK inhibitors (Kawasaki et al., 2004). In line with these data, CGRP signaling pathways activate CREB-mediated P2X3 receptor expression and function (Simonetti et al., 2008).

Using a transgenic knock-in (KI) mouse exhibiting Ca_V_2.1 R192Q mutated voltage-gated calcium channels (P/Q-type) (Tottene et al., 2009), we previously identified multiple interactors (calcineurin, Cyclin-dependent kinase 5 (Cdk5) and CaMKII) associated to the gain of function of the mutated channel leading to larger intracellular calcium levels that modulate P2X3 receptor function in trigeminal sensory neurons. In particular, enhanced P2X3 receptor-mediated responses are found in KI neurons that depend on constitutive activation of CaMKII and are reversed by the selective Ca_V_2.1 channel blocker ω-Agatoxin or by pharmacological block of CaMKII (Nair et al., 2010a). CaMKII sensitivity to intracellular calcium levels, is an important switcher of different intracellular pathways (i.e., Cdk5) that influence P2X3 receptor activity and function, as demonstrated in mice expressing Ca_V_2.1 mutated channels (Nair et al., 2010a). CaMKII is also involved in P2X3 receptors export towards the surface membrane (Xu and Huang, 2004; Fabbretti et al., 2006; Hasegawa et al., 2009), a process that is largely dependent on ambient temperature (Pryazhnikov et al., 2011). Furthermore, the typical agonist-evoked desensitization of P2X3 receptors is associated to dynamic, calcium-sensitive redistribution of such receptors to lipid raft domains (Vacca et al., 2009; Gnanasekaran et al., 2011) and internalization (Vacca et al., 2011; Chen et al., 2012). Thus, the intracellular calcium homeostasis is important to modulate P2X3 receptor responses, as the calcium sensor neuronal Ca2+−sensor proteins (VILIP1) forms a signaling complex with P2X receptors and regulates P2X3 receptor sensitivity to ATP, and it even enhances the neuronal excitability of naive dorsal root ganglion (DRG) neurons (Chaumont et al., 2008; Liu et al., 2013).

In order to transduce ATP signals to downstream responses, we hypothesize that P2X3 receptors require discrete sorting to membrane compartments where, on a short term on-demand basis, all the molecular elements necessary for the correct signal transduction are anchored. Among many, calcium/calmodulin-dependent serine protein kinase (CASK) is a scaffold protein of emerging importance (Hsueh, 2011), sensitive to intracellular calcium, CamKII levels (Lu et al., 2003; Hodge et al., 2006; Malik et al., 2013) and Cdk5 (Samuels et al., 2007), all elements known to strongly modulate P2X3 receptors (Nair et al., 2010a,b).

**Figure 1:**
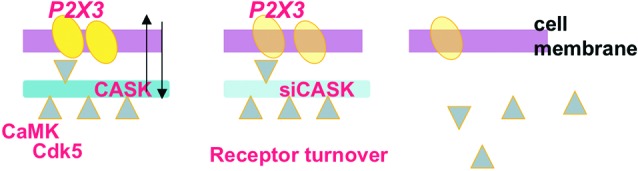
**Scheme of dynamic assembly of the CASK/P2X3 receptor complex at neuronal membrane level.** The scaffold protein CASK (blue; left) and the P2X3 receptor (yellow) are associated in the same macromolecular complex. Note that adaptor molecules like CamKII and Cdk5 (triangles) are proposed to regulate the CASK/P2X3 receptor complex at membrane level and determine the strength of their interaction. Silencing CASK (middle and right panels) results in uncoupling of the CASK/P2X3 receptor complex followed by internalization of P2X3 receptors and their proteasomal degradation, suggesting that CASK is the anchor to maintain P2X3 receptor at membrane level.

**Figure 2:**
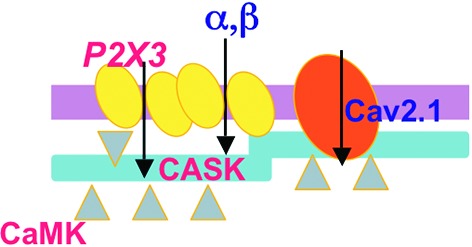
**Scheme of the CASK/P2X3 receptor complex in the R192Q mutation of the cacna1a gene.** Both CASK and P2X3 receptors are more expressed in membrane lipid rafts of missense Cacna1a KI neurons, suggesting a role of CASK in creating larger P2X3 receptor clusters. Ca_V_2.1 R192Q channel gain of function and enhanced CamKII activity produced by the increased influx of calcium are important for the formation of the CASK/P2X3 complex and receptor function.

CASK and P2X3 receptors are found within the same macromolecular complex: our data suggest that CASK acts like a docking point to stabilize P2X3 receptors expression at membrane level, as the CASK knockdown results in proteasome-dependent receptor disassembly and reduced P2X3 receptor current (Figure 1; Gnanasekaran et al., 2013). Interestingly, CASK is typically more directed to lipid rafts (Gnanasekeran and Fabbretti, unpublish data) and more strongly associated with P2X3 receptors in the voltage-dependent P/Q-type calcium channel subunit alpha-1A (CACNA 1A) KI mice (Pietrobon, 2010), characterized by altered calcium channel and CamKII activity (Figure 2; Gnanasekeran et al., under revison). In the KI model, CASK/P2X3 complex is uncoupled by *ω*-Agatoxin or the CaMKII inhibitor KN-93, reinforcing the role of intracellular calcium in the modulation of P2X3 receptors in sensory neurons.

**Figure 3:**
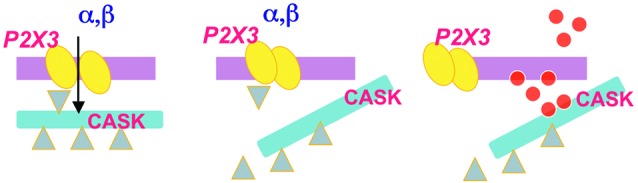
**Idealized diagram of the dynamic nature of the CASK/P2X3 complex.** P2X3 receptor agonist application (*α*,*β*) induces an inward cationic current (left panel) that requires correct assembly of CASK/P2X3. When the agonist application is sustained to produce receptor desensitization, disassembly of the CASK/P2X3 complex occurs (middle). Thus, untethered CASK can be redirected to distinct downstream signaling (right panel) via multiple effectors (red dots).

One of the peculiar findings associated with the CASK/P2X3 complex is its dynamic nature that largely depends on the receptor functional activity (Figure 3; Gnanasekaran et al., 2013). In particular, nociceptive stimulation with NGF application strengthens P2X3/CASK co-purification, while P2X3 receptor function is sufficient to dissociate the complex (Gnanasekaran et al., 2013). It is, therefore, likely that both P2X3 receptor activity and CASK regulators (as CaMKII) control the CASK/P2X3 complex. In its role as scaffold protein, CASK links different adaptors and molecules (including other channels) to elicit further downstream signaling, like the stability and trafficking of receptors towards the membrane (Hsueh, 2011) and vesicle release (Spangler et al., 2013). In brain synapses, CASK has a negative role, as facilitated glutamatergic release was observed in KO CASK mice (Atasoy et al., 2007), in agreement with the inhibitory effect of CASK over the P2X3 over-reactivity (Gnanasekaran et al., 2013).

Recent findings suggest that CASK, known to modulate export and trafficking of N-methyl-D-aspartate (NMDA) receptors (Jeyifous et al., 2009), induces synapse-associated protein 97 (SAP97) conformation changes to control the rate of glutamate receptor insertion into the synaptic compartment (Lin et al., 2013). Whether a similar process occurs in sensory ganglia or at central synapse, and if ATP has a role in this modulation remain a matter for future studies.

It is possible that altered CASK targeting in chronic pain states could impair communication between satellite cells and neurons via aberrant gap junction/hemichannels function (Márquez-Rosado et al., 2012) and with possible damage of neuro-satellite cell units. In particular, binding of CASK to neurexins and neuroligins in heterologous synapses (Fairless et al., 2008; Gokce and Südhof, 2013), indicates its potential involvement in a structural process to shape the extent and location even of neuron/non-neuronal cell communication.

Furthermore, at central level, it seems likely that presynaptic CASK/P2X3 functional interaction regulates synaptic strength in the spinal dorsal horn, reinforcing the interest for P2X3 receptors as key modulators of the fiber sensitivity in chronic pain.

## Conclusions

In view of their high agonist affinity, P2X3 receptors appear as major candidates to sense even small changes in extracellular ATP and transduce them into downstream neuronal responses. Further potentiation of the ATP effects is determined by intracellular calcium signaling and subsequent kinase activation that mediates P2X3 receptor phosphorylation, expression and turn-over. Finally, membrane specialized domains could convey specific responses via dedicated signal transduction machinery whereby CASK could serve as a platform to orchestrate ATP signaling through sorting and redistribution of P2X3 receptors. ATP receptor stimulation could determine further release of neuromodulators (Gu and MacDermott, 1997), recruitment of inflammatory cells and progression to neuronal sensitization, thus contributing to fine regulatory mechanisms and strong plasticity of sensory neurons. Understanding the spatio-temporal scale of these processes is a priority to propose molecular targets useful for clinical applications to chronic pain.

## Conflict of interest statement

The authors declare that the research was conducted in the absence of any commercial or financial relationships that could be construed as a potential conflict of interest.
